# Marking through molts: An evaluation of visible implant elastomer to permanently mark individuals in a lower termite species

**DOI:** 10.1002/ece3.8030

**Published:** 2021-08-18

**Authors:** Rebecca F. B. Padget, Faye J. Thompson

**Affiliations:** ^1^ Centre for Ecology and Conservation College of Life and Environmental Sciences University of Exeter Cornwall UK; ^2^ Centre for Research in Animal Behaviour College of Life and Environmental Sciences University of Exeter Exeter UK

**Keywords:** hemimetabolous insects, individual marking, social insects, termites, visible implant elastomer

## Abstract

1. Advances in individual marking methods have facilitated detailed studies of animal populations and behavior as they allow tracking of individuals through time and space. Hemimetabolous insects, representing a wide range of commonly used model organisms, present a unique challenge to individual marking as they are not only generally small‐bodied, but also molt throughout development, meaning that traditional surface marks are not persistent.
2. Visible implant elastomer (VIE) offers a potential solution as small amounts of the inert polymer can be implanted under the skin or cuticle of an animal. VIE has proved useful for individually marking fish, crustaceans, and amphibians in both field and laboratory studies and has recently been successfully trialed in laboratory populations of worms and fly larvae. We trialed VIE in the single‐piece nesting termite *Zootermopsis angusticollis*, a small hemimetabolous insect.
3. We found that there was no effect of VIE on survival and that marks persisted following molting. However, we found some evidence that marked termites performed less allogrooming and trophallaxis than controls, although effect sizes were very small.
4. Our study suggests that VIE is an effective technique for marking small hemimetabolous insects like termites but we advocate that caution is applied, particularly when behavioral observation is important.

## Graphical abstract

Marking individuals allows detailed studies of behavior and life history, but permanently marking individuals in species that undergo molts is notoriously difficult. Here we use visible implant elastomer (VIE) to mark individuals in a species of termite, *Zootermopsis angusticollis*. We show that marking using VIE has little effect on survival or behavior and, importantly, VIE marks persist through molts.

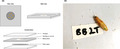

## 1 INTRODUCTION

Individually marking animals is a useful tool in the study of animal populations, ecology, and behavior in both field and laboratory research (Hjort & Lindholm, 1978). Persistent marks are particularly useful for animals that are difficult to track by an experimenter, for example, because they are regularly obscured by the environment, live in inaccessible areas or because animals look similar to each other. These difficulties necessitate the use of identifying marks that are reliably persistent, but marks must also be noninvasive such that they do not impact individuals’ survival or behavior (Batsleer et al., 2020).

Recent advances in tracking software and machine learning algorithms have facilitated automated high‐throughput data collection of unmarked animals, including insects (Imirzian et al., 2019). While these methods are likely to prove invaluable for large‐scale behavioral studies, individual identities cannot generally be maintained, meaning that medium‐ and long‐term studies are likely to still require individual marking. Hemimetabolous insects present a unique combination of challenges to consider when applying long‐lasting marks. Principally, hemimetabolous insects molt during development, meaning that surface marks widely used on other insects—such as paint or correction fluid—only persist until the next molt, which is often difficult to predict in standard natural or laboratory populations. This makes medium‐ or long‐term studies of individuals challenging as surface marks would require regular reapplication, and it could be difficult or impossible to reidentify individuals that have lost their marks. To combat this issue, oil‐soluble dyes that collect in insect soft tissues have been used for internally marking insects (Hagler & Jackson, 2001; e.g., in Vilarinho et al., 2006). These dyes can be persistent, though their efficacy can be dependent on environmental conditions such as diet (Thorne et al., 1996). Internal dyes can also be transferred among individuals both vertically and horizontally via trophallaxis and other social interactions (Hagler & Jackson, 2001; e.g. da Silva Camargo et al., 2017), which means that they cannot be used to reliably mark individuals, particularly of social species. Some of these dyes also require specialist equipment like UV lights, while others rely on dissecting the insects to see the color internally, eliminating the possibility of continued observation (Schroeder & Mitchell, 1981).

Hemimetabolous insects are generally small‐bodied animals, meaning that even a small mark can be relatively invasive, potentially affecting survival and behavior, and having both ethical and scientific implications (Batsleer et al., 2020; De Souza et al., 2012). Some marks, such as surface paints, could also affect behavior because the volatile chemicals they contain can interfere with chemical communication, which is important in many insect species (Jürgens & Bischoff, 2017; Wang et al., 2016). It is therefore a challenge to mark small insects in a way that is both reliably long‐lasting and noninvasive.

Visible implant elastomer (VIE; Northwest Marine Technology, Inc, Anacortes, USA) is used to mark individuals by injecting a small bead of inert, colored polymer under the skin or cuticle. VIE has been widely used in both natural and laboratory populations of reptiles, amphibians, and fish (Bainbridge et al., 2015; Bushon et al., 2007; Penney et al., 2001). VIE has also been trialed in blow flies and earthworms, with results suggesting no impact on survival or development (Butt & Lowe, 2007; Moffatt, 2013). Because VIE is implanted under the cuticle, it should persist through the molts of hemimetabolous insects and should not disrupt chemical communication. Additionally, small, controlled amounts of the polymer can be injected with a microneedle, meaning that even small individuals can be marked.

In this study, we examine VIE as a new method of permanently marking individuals of hemimetabolous insect species by testing its potential effects on survival and behavior in termites. Termites are commonly used in studies of ecology and evolution, for example, in studies of social evolution (Johns et al., 2009), host–symbiont coevolution (Noda et al., 2007), social immunity (Rosengaus et al., 1998), and collective behavior (Miramontes & DeSouza, 2008). Termites, representing the earliest transition to eusociality, provide an ideal system in which to study the evolution of sociality in general and of eusociality specifically (Korb, 2009; Korb & Heinze, 2016). The phylogenetically basal “lower” termite species are particularly valuable for understanding the roles of individual‐ and group‐level selective forces in the evolution of sociality as they retain some ancestral behavior—the workers of many of these species retain the capacity to become reproductive throughout life (Korb & Heinze, 2016). By contrast, workers of “higher” termite species (Termitidae; Korb, 2007), as in other eusocial Hymenoptera, are permanently sterile. Despite these behavioral differences between higher and lower termites, the application of VIE is likely to be useful to both since both are hemimetabolous (to some extent) and undergo transitional and developmental molts.

We conducted this trial with the single‐piece nesting lower termite species, *Zootermopsis angusticollis* (Hagen, 1858) Termopsidae. In *Z. angusticollis* and other single‐piece nesting species, a monogamous reproductive pair founds a colony on a single piece of wood, which is the food source and nesting material of the colony. Each colony comprises a reproductive royal pair, and numerous workers and soldiers. Workers are developmentally plastic and totipotent: They can differentiate (via molts) into sterile soldiers, or into a reproductive form, either to remain in their natal nest as a secondary reproductive, or to disperse to found a new nest. The ability of individuals to follow different development trajectories means that marking individuals to track them through their life is particularly important.

We tested VIE in a randomized trial in our laboratory population of *Z. angusticollis*. We censused small groups of termites that had been injected with VIE alongside noninjected control groups and conducted behavioral assays to establish whether VIE affected survival or behavior of laboratory‐cultured termites.

## 2 METHODS

### 2.1 Colony collection and maintenance

Thirteen natural colonies of *Z*. *angusticollis* (each containing approximately 1,000 individuals) were collected with their log nest material from redwood parks in California, USA (5 colonies from Redwood Regional Park (37°48′49″N, 122°09′57″W); 3 colonies from Henry Cowell Redwoods State Park (37°2′22″ N, 122°3′49″ W); 2 colonies from Big Basin Redwoods State Park (37°10′23″N, 122°13′17″W); 1 colony from Butano State Park (37°12′08″N, 122°20′22″W); 1 colony from Forest of Nisene Marks State Park (36°59′11″N, 121°54′16″W); 1 colony from Sugarloaf Ridge State Park (38°26′31″N, 122°30′49″W)). Colonies were collected and exported under permit from East Bay Regional Parks, California Department of Parks and Recreation, and the California Department of Fish and Wildlife, and imported to the Centre for Ecology and Conservation, University of Exeter, UK, under license from the UK Animal and Plant Health Agency. Colonies were collected in May 2018 (5 colonies) and June 2019 (8 colonies) and were maintained in 35 liter plastic boxes in a controlled environment room at 22℃ and 85% humidity and in darkness. Colonies were sprayed with distilled water twice weekly to maintain humidity and replenished ad libitum with silver birch wood (*Betula pendula*). Experimental trials were carried out between January and March 2020.

### 2.2 Experimental design

**FIGURE 1:**
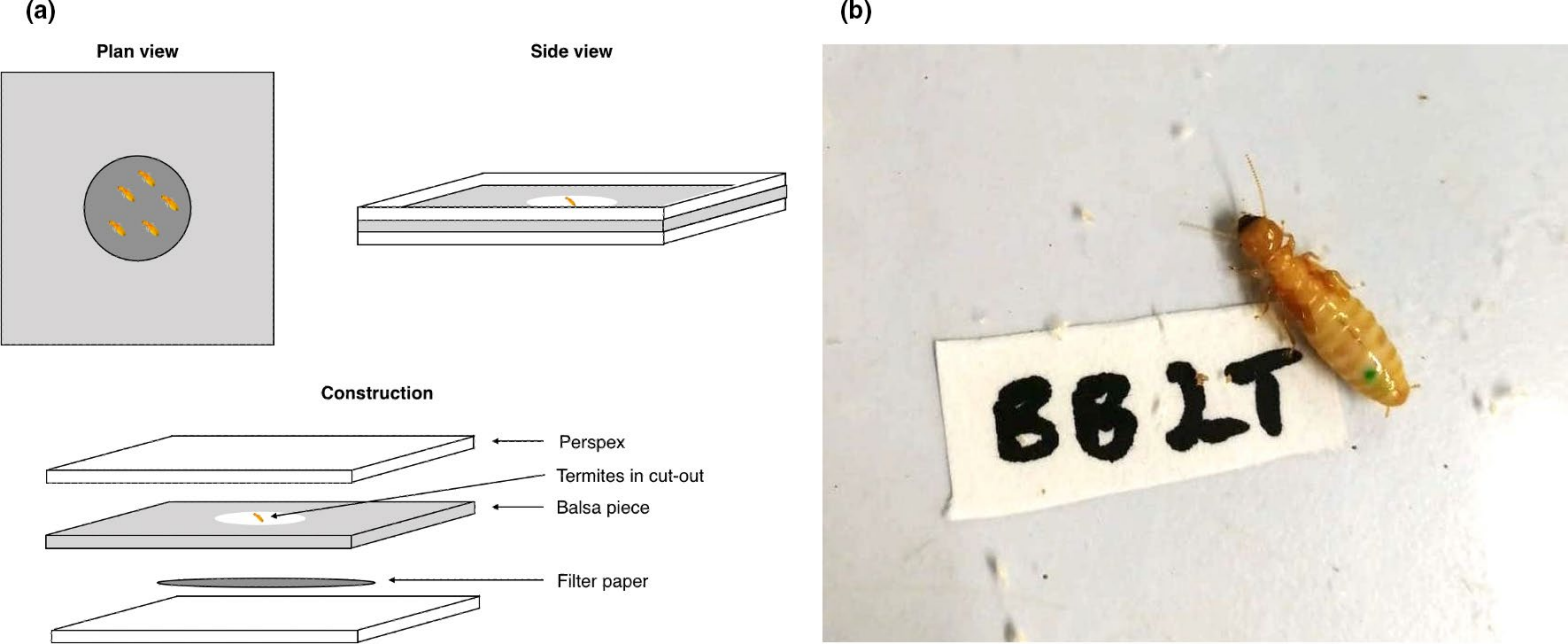
Schematic of balsa wood housing (a) and photograph of a marked termite showing placement of the VIE implant (b). (a) Balsa was placed in between two Perspex sheets and the termites placed in a cut‐out circle in the middle of the wood. (b) Photograph shows the placement of the VIE (green mark) on the upper abdomen of a termite nymph

We extracted ten termites (adults, identified by their sclerotized exoskeleton; Noirot & Pasteels, 1987) from each colony (*N* = 130 individuals; 70 pseudergates and 60 nymphs with developed wing buds) and randomly assigned each termite to a treatment or control group (5 termites per group; *N* = 13 treatment and 13 control groups). Males and females of *Z. angusticollis* are similar in morphology and behavior at the pseudergate and nymph stage; we therefore did not note the sex of the extracted termites. Each group was transferred to its own experimental housing. This housing comprised a single 3 mm‐thick piece of balsa wood 120 mm square with a 45 mm‐diameter circle cut out of the center into which termites were placed. The balsa was then sandwiched between two sheets of 2 mm clear, colorless Perspex (also 120 mm square) with a sheet of 50 mm‐diameter cellulose filter paper moistened with distilled water (Figure 1a); the construction was fastened using bulldog clips. This set up enabled us to view termites for the duration of the trial and to perform censuses and behavioral assays without disturbance. Termite housings were stored in a single layer in flat eight‐liter plastic boxes lined with moistened paper towel, sprayed with distilled water twice weekly to maintain moisture, and kept in a controlled environment room in the dark at 22℃ and 85% humidity. If termites began to tunnel toward the edge of the balsa wood, a small amount of metal gauze was clipped to the open edge to prevent escape.

Termites were left to acclimatize to their housing for 24 hr. The following day, R.P. performed the VIE implant procedure to termites in each treatment group while F.T. simultaneously performed the same experimental procedure to termites in each equivalent control group (from the same original colony) except that control termites were not injected. This ensured that any effects we observed were a result of the injection and VIE implant, rather than disturbance or handling. For each treatment‐control group pair, we extracted one termite from each group and cold‐immobilized them by placing them in separate compartments of an empty ice cube tray placed on ice for ~20 s (or until movement ceased). The pair of termites were removed from the ice cube tray, and the treatment termite was injected with a small dot of green VIE, prepared by mixing a drop of setting agent with ~0.2 ml of the color component in a manual syringe with a 29‐gauge (0.34 mm outer diameter) needle (supplied in the VIE kit by Northwest Marine Technology). The VIE was injected under the cuticle, through the intersegmental membrane, on the upper abdomen (Figure 1b). We did not use magnification during injection. The control termite was handled in the same way for the time that it took to insert the VIE implant in the treatment termite. Termites were held securely between the first two fingers and thumb to prevent movement and provide access to the upper abdomen. Once the VIE had been implanted, each termite was returned to its respective housing.

### 2.3 Survival and behavioral data collection

Termites were kept in their housing and censused daily for 35 days, following which they were censused weekly for a further four weeks, totaling a 63‐day trial period. At each census, we recorded the number of termites alive in each treatment and control group. Any termites that died during this period were left in their housing to avoid differences in disturbance levels among groups.

To collect behavioral data, groups were filmed for fifteen minutes on days 2, 4, and 10 after treatment. If termites had created tunnels which would obscure them in the video, the top wood of this tunnel was cut away with scissors, otherwise termites remained undisturbed. Each group was filmed for 15 min in its housing. Videos were recorded under red light in an otherwise darkened room—two red light bulbs were positioned roughly 1.5 m above the termite housing. A Sony HDR‐CX240E or a Sony HDR‐PJ330 video camera was mounted on a tripod, roughly 30 cm above the housing to record a single group. Videos were coded at 4× speed by focal sampling each termite using Behavioural Observation Research Interactive Software (BORIS; Friard & Gamba, 2016). Data were only collected from the last ten minutes of each videoing period to give the termites a five‐minute acclimatization period so as to ensure behavior was not affected by the disturbance of being moved from their storage boxes.

We measured six behaviors: allogrooming, antennation, butting, self‐grooming, trophallaxis, and any interaction with the environment—that is, the wood, filter paper, or feces in the housing (Table 1). For allogrooming, antennation, trophallaxis, and manipulation of the environment, we measured the proportion of time that each behavior was performed; for butting and self‐grooming, we recorded the number of occurrences because both butting and self‐grooming occurred in short bursts with little discernible variation in the length of time that each event lasted.

**TABLE 1:** Behaviors measured during video observation

Behavior	Description	Data type	References
Allogrooming	Focal termite uses mandibles on other termite's body or head such as not to cause injury or flee response from the recipient	Proportion time spent performing behavior	Korb (2008) Korb et al. (2012) Zhukovskaya et al. (2013)
Antennation	Focal termite moves antennae over other termite's head, body, or antennae	Proportion time spent performing behavior	Korb et al. (2012)
Butting	Quick vibrations of the whole body by focal termite	Count	Korb et al. (2012)
Environment manipulation	Focal termite uses mandibles to chew filter paper or wood, or move pieces of filter paper, wood, or feces around the housing	Proportion time spent performing behavior	–
Self‐grooming	Focal termite pulls antenna forward to mandibles	Count	Rosengaus and Traniello (1991, 1993) and Wilson‐Rich et al. (2007)
Trophallaxis	Focal termite has mouth to mouth or mouth to anus contact with another termite	Proportion time spent performing behavior	Crosland et al. (1997) (but see Korb and Schmidinger, 2004)

From videos, we identified recently molted individuals by their paler heads and mandibles. For treatment termites, we noted whether the VIE mark was still visible.

### 2.4 Ethics

Termite colonies were collected with permission from the United States Department of Agriculture (permit number P526P‐ 17‐03814 issued to Rebeca B. Rosengaus, Northeastern University, Boston), the State of California Department of Fish and Wildlife (permit number 13290), the East Bay Regional Park District (permit number 977), and the California Department of Parks and Recreation (permit number 19‐820‐31). This research was approved by the College of Life and Environmental Sciences (Penryn) ethics committee (application ID: eCORN002610 v2.1) and carried out in accordance with the guidelines set out by the Association of the Study of Animal Behaviour for the treatment of animals in research.

### 2.5 Statistical analyses

#### 2.5.1 Survival analysis

A total of 130 termites were initially extracted from colonies but one control was injured during transfer to the arena on day 0 and so was removed from analysis, leaving 64 control individuals and 65 treatment (VIE‐injected) individuals housed in 26 groups. To model survival over time, we used census data to generate Kaplan–Meier survival curves and performed a log‐rank test to test the null hypothesis that survival distributions were the same in the treatment and control groups. We separately modeled survival over the entire 63‐day sampling period and over the first ten days to identify any short‐term effects that might have been obscured over a longer time frame.

To investigate factors that affected the hazard (the instantaneous probability of death), we used a mixed effects Cox proportional hazards model with experimental group (treatment or control) as a fixed effect with unique housing ID (1–26) nested within colony of origin as a random effect. Here, we report the estimated coefficient, the hazard ratio (HR) and the 95% confidence interval of the hazard ratio, and the *p*‐value. We fitted a Cox proportional hazards model to the data over the entire 63‐day census period.

To validate the fit of our Cox proportional hazards model, we simulated time until death data for 130 individuals in 1,000 simulated trials using the Cox model coefficient, and deaths per day per individual (calculated from the data) as a basic linear hazard function.

Analyses were carried out in R 3.6.1 using the “survival” and “survminer” packages (Kassambara et al., 2019; Therneau & Grambsch, 2000).

#### 2.5.2 Behavioral analysis

We conducted principal component analysis to investigate whether the six behaviors measured could be reduced to fewer underlying behavioral patterns and to identify any clustering in the data by either experimental group (treatment or control) or day after treatment (2, 4, or 10, as factors).

Data for all behaviors were zero‐inflated and, as such, the assumptions of standard linear models were not met. This was not improved by transformation of the data. We therefore used a Bayesian framework to fit generalized linear mixed effects models (GLMMs) to data for each behavior with Markov chain Monte Carlo (MCMC) and weakly informative priors ($\beta_{i}∼N\left(0,2.5\right)$). We fitted models to untransformed data, assuming a zero‐inflated beta distribution for the proportion time data (allogrooming, antennation, environment manipulation, trophallaxis; Douma & Weedon, 2019) and a negative binomial for the count data (butting and self‐grooming). We included experimental group (treatment or control) and day after treatment (2, 4, and 10, as a categorical variable) and their interaction as fixed effects, and with unique housing ID (1–26), nested within colony of origin as random effects. Models were fitted to a total of 369 focal observation periods in 78 videos over the three videoing days. On day 2, 124 individuals were sampled (treatment: *n* = 60, control: *n* = 64); on day 4, 123 individuals were sampled (treatment: *n* = 59, control: *n* = 64), and on day 10, 122 individuals were sampled (treatment: *n* = 58, control: *n* = 64).

To test the effect of each predictor, we calculated its inclusion Bayes factor. The inclusion Bayes factor for a predictor is the ratio of the average posterior likelihood of models including the predictor of interest over that of models excluding the predictor of interest (van den Bergh et al., 2020). We restricted the pool of models to test such that models containing interaction terms without their main effects were not included (Franzese & Kam, 2009). Following convention set out in Jeffreys (1961; see also Jarosz & Wiley, 2014; Lee & Wagenmakers, 2014), we interpret predictors to have moderate support if the inclusion Bayes factor is between three and 10, strong support if it is between 10 and 30, very strong support between 30 and 100, and extreme support for inclusion Bayes factors above 100.

To find effect sizes for each term, we calculated Bayes factors based on the posterior likelihood of each model (i.e., produced by removing predictors in turn) against the null (intercept only) model. We calculated effect sizes from the model with the highest support from the Bayes factor even if inclusion Bayes factors were less than three (indicating negligible support). We calculated effect sizes from the selected models by back‐transforming model parameters using the inverse of the link functions used to fit each model. For the models of proportion data (allogrooming, antennation, environment manipulation, and trophallaxis), we used the logistic function—the inverse of the logit link function; for the count data (butting, self‐grooming), we used the exponential (the inverse of the log link function). Since the effect size considered acceptable for a given study will depend on the application of VIE, we report the median of the effect size distribution from our models and 89% highest posterior density intervals (HDI). The 89% HDI gives the region within which 89% of the density of the distribution lies. This quantifies the uncertainty around the estimate to allow researchers to evaluate whether VIE is an acceptable tool for their specific study.

We used the “brms” package for the zero‐inflated beta models (Bürkner, 2018) and the “rstanarm” package for the negative binomial models (Brilleman et al., 2018). We used “bayesplot” (Gabry et al., 2019) and “bayestestR” (Makowski et al., 2019) for analysis and visualization of models and “ggplot2” to produce figures (Wickham, 2016).

## 3 RESULTS

### 3.1 Survival analysis

Of 64 control individuals, 49 (77%) survived until the last census day (day 63). Of 65 treatment (VIE‐injected) individuals, 42 (65%) survived until the last census day. The mean number alive in each housing on the final day of censusing was 3.8 for the controls and 3.2 for the treatment groups (Table S1), however, there was large variation that suggested nonindependence among individuals in the same housing.

**FIGURE 2:**
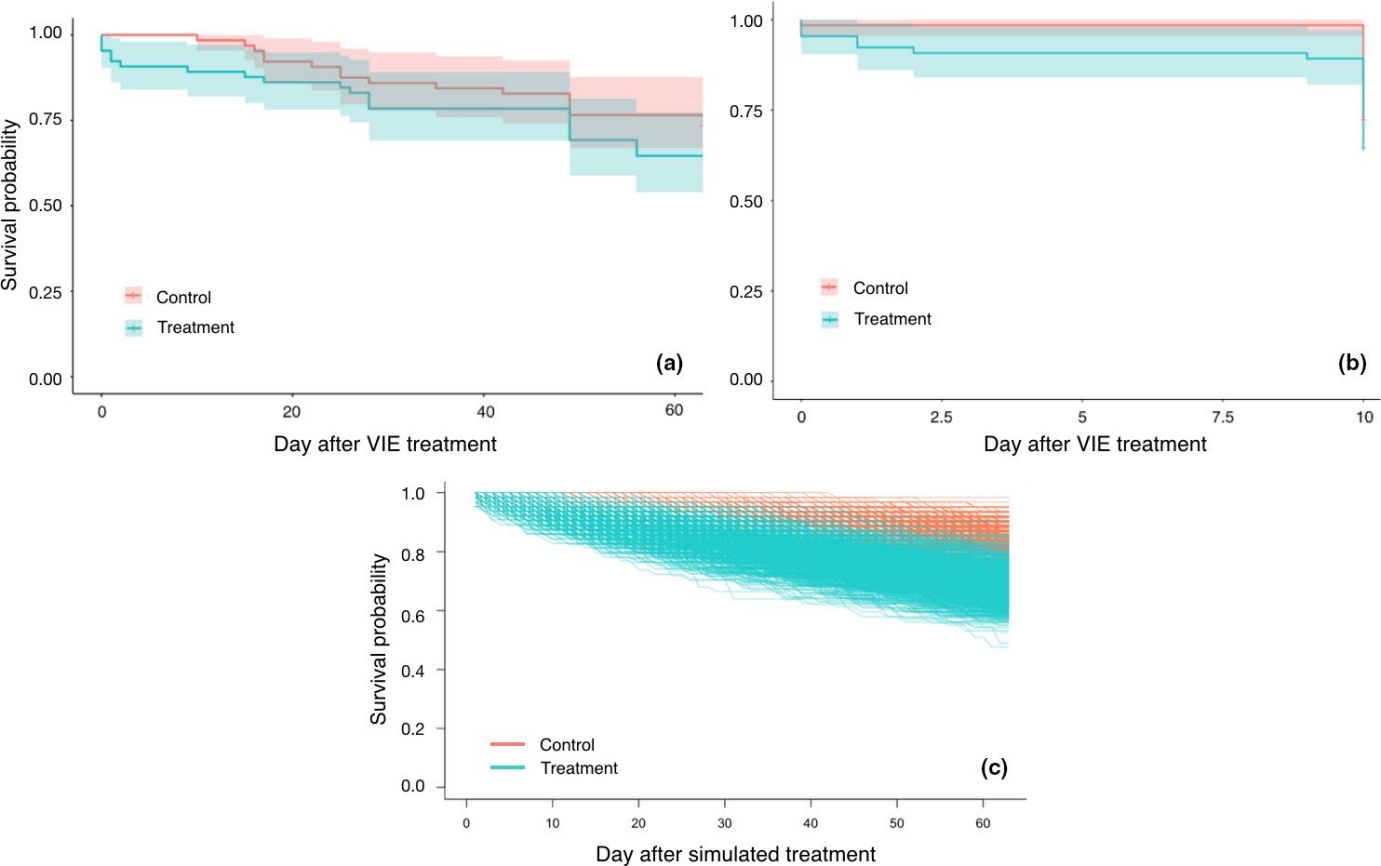
Kaplan–Meier survival curves for treatment and control groups. Plots show survival curves and 95% confidence intervals using observed survival data for treatment (blue) and control (red) groups for (a) the entire 63‐day census period, and (b) the first ten days of censusing. Survival curves using simulated survival data for treatment (blue) and control (red) groups are shown in (c)

Survivorship of the treatment and control groups was not significantly different over the full 63 days of the experiment (log‐rank test: χ^2^ = 1.3, *p* = .26; Figure 2a) or during the first 10 days (log‐rank test: χ^2^ = 1.4, *p* = .24; Figure 2b).

We also found no significant difference in the hazard of treatment and control groups (mixed effects Cox proportional hazards model: β = 0.85, HR = 2.3, 95% CI = −0.94, 2.6, *p* = .35). The hazard ratio of 2.3 indicated a roughly twofold increase in probability of death on any given day in treatment compared to control termites. However, the 95% confidence interval was large (and spanned zero), suggesting a high level of variation in survival times within both groups, likely as a result of the low overall number of deaths that we observed. Our simulated data produced survival curves that were significantly different between the treatment and control group in 60% of trials (598 out of 1,000 simulations; Figure 2c), given the Cox model coefficient of 0.85 and an alpha value .05. Given that the Cox model coefficient is nonzero, this result suggests that there was relatively low statistical power, likely as a result of the low number of deaths that we observed overall.

### 3.2 Behavioral analysis

Principal component analysis showed that the observed levels of the behaviors we measured (allogrooming, antennation, butting, environment manipulation, self‐grooming, and trophallaxis) did not cluster by experimental group or by day after treatment (Figure S1). The first two principal components explained only 21.8% and 17.9% of the variation, suggesting that there were no common drivers underlying the behaviors that we observed. We therefore conducted separate analyses for each behavior.

Inclusion Bayes factors for models are shown in Table 2. Inclusion Bayes factors showed moderate to strong support for an effect of treatment on allogrooming (BF_inc_ = 10), and moderate support for an effect of treatment on antennation (BF_inc_ = 6.1) with treatment individuals performing less allogrooming and more antennation. There was also strong support for an interaction between treatment and day for trophallaxis, whereby control individuals increased their rate of trophallaxis on day 10, but treatment individuals did not (BF_inc_(treatment:day) = 11; BF_inc_(day) = 4.3; BF_inc_(treatment) = 2.2). We found no support for an effect of treatment on either environment manipulation (BF_inc_ = 0.69) or self‐grooming (BF_inc_ = 1.0), and we found evidence against an effect of treatment for butting (BF_inc_ = 0.25).

**TABLE 2:** Inclusion Bayes factors for each predictor in the model for each behavior

Behavior	Model	BF_inc_
Allogrooming	Treatment	10
	Day	4.5
	Treatment × Day	0.73
Antennation	Treatment	6.1
	Day	0.28
	Treatment × Day	0.41
Environment manipulation	Treatment	0.69
	Day	3.2
	Treatment × Day	1.4
Trophallaxis	Treatment	2.2
	Day	4.3
	Treatment × Day	11
Butting	Treatment	0.25
	Day	64
	Treatment × Day	0.66
Self‐grooming	Treatment	1.0
	Day	0.36
	Treatment × Day	0.35

We found evidence that butting and allogrooming were increased on day 10, independent of treatment (butting BF_inc_ = 64; allogrooming BF_inc_ = 4.5). Environment manipulation was lower on day 4 for both groups (BF_inc_ = 3.2). There was no evidence for an effect of day on self‐grooming (BF_inc_ = 0.36), and moderate evidence against an effect of day on antennation (BF_inc_ = 0.28).

Bayes factor comparisons of models against the null model were qualitatively in agreement with the inclusion Bayes factors. Models with the highest Bayes factors (i.e., those with the best fit to the data) included predictors that received some support from the inclusion Bayes factors. Bayes factors for models of butting and self‐grooming were all less than 3, suggesting that variation was not well‐captured by any of the models. To ensure that no effects were missed, we used the butting and self‐grooming models with the highest Bayes factors to calculate effect sizes, despite the inclusion Bayes factors showing no support for an effect.

**FIGURE 3:**
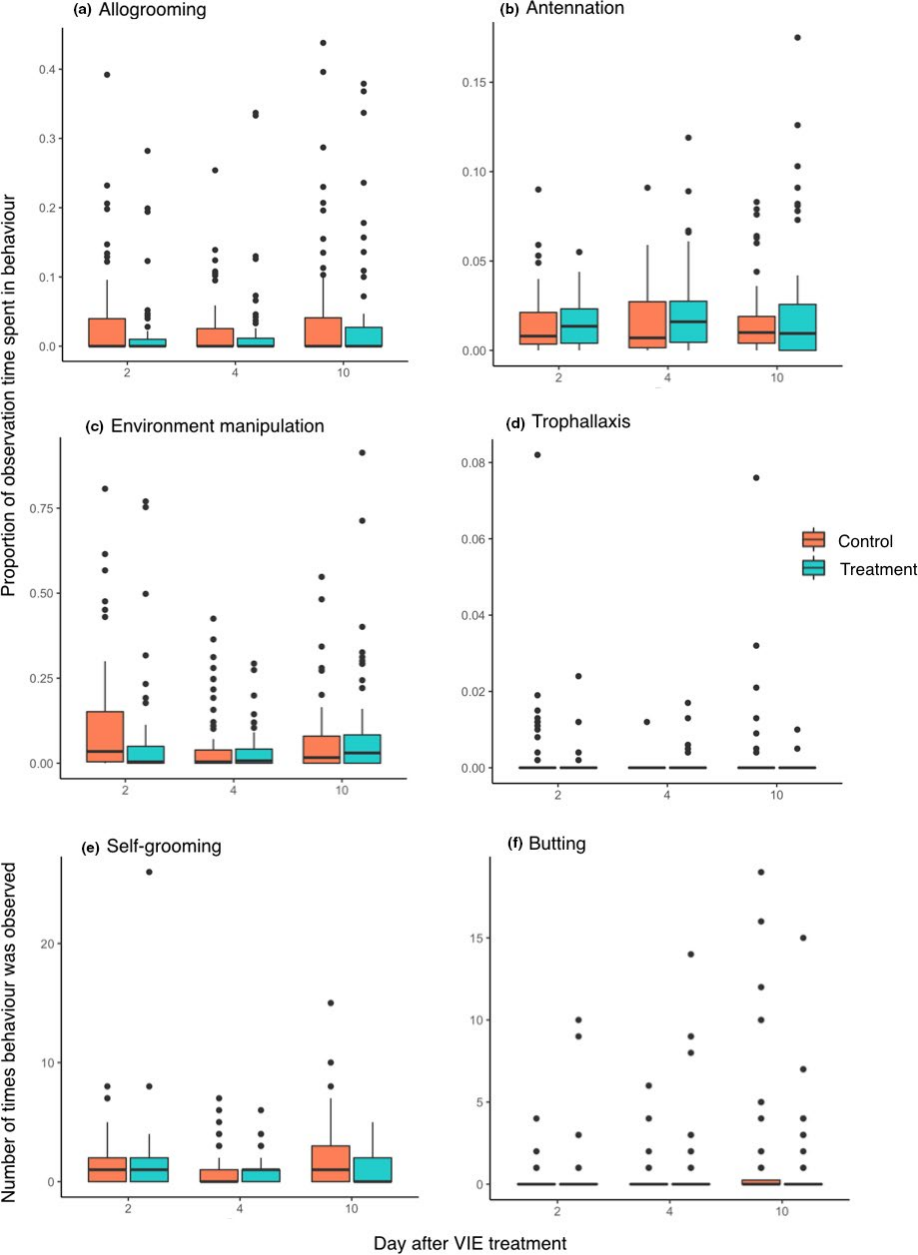
The effect of VIE marking on behavior. Box‐and‐whisker plots of raw data for (a) allogrooming, (b) antennation, (c) environment manipulation, (d) trophallaxis, (e) self‐grooming, and (f) butting at days 2, 4, and 10 after VIE treatment for control (red) and treatment (blue) groups. Boxes represent the interquartile range, the median is shown by the horizontal black line, and the vertical lines represent 1.5 times the interquartile range. Points show data that falls outside of this range

Median effect sizes for all predictors were between −0.05 and +0.05 difference in proportion time spent performing the behavior for allogrooming, antennation, environment manipulation, and trophallaxis and represented an increase or decrease of less than one event over the ten‐minute observation period for butting and self‐grooming (Table 3; Figures S2–S7). Plots of raw data for each behavior show comparatively little difference between control and treatment individuals across the experimental period (Figure 3).

**TABLE 3:** Median effect size and 89% highest posterior density interval for each predictor in the model for each behavior

Behavior	Predictor	Median effect size	89% HDI
Allogrooming	Treatment	−0.023	−0.04, −0.01
	Day 4	0.0033	−0.02, 0.02
	Day 10	0.035	0.02, 0.06
Antennation	Treatment	0.0042	0, 0.01
Environment manipulation	Day 4	−0.029	−0.05, 0
	Day 10	−0.0012	−0.03, 0.02
Trophallaxis	Treatment	−0.00085	−0.01, 0.01
	Day 4	0.0018	−0.02, 0.04
	Day 10	0.013	0, 0.03
	Treatment:Day 4	9.3e‐5	−0.05, 0.03
	Treatment:Day 10	−0.012	−0.05, 0.01
Butting	Day 4	0.083	−0.02, 0.19
	Day 10	0.29	0.15, 0.43
Self‐grooming	Treatment	−0.038	0, 0.01

### 3.3 Persistence of the VIE mark

By day 2 (the first day of behavioral assay filming), two termites in two different treatment groups had molted. We identified molted individuals in these videos as they had a noticeably paler body and mandibles, and there was a shed exoskeleton in the housing. On both molted individuals, the VIE mark remained clearly visible (Figure S8). Videos recorded on day 10 show that 52 of 58 surviving termites (90%) had retained their VIE marks. Of the six individuals (in four treatment groups) that lost marks, one individual lost its mark by day 2 and the remaining five lost their mark between days 4 and 10. None of the individuals that lost their marks showed any signs of having recently molted, nor was there a shed exoskeleton present in their housing.

## 4 DISCUSSION

We investigated the effect of visible implant elastomer (VIE) implants on the survival and behavior of *Z*. *angusticollis* to evaluate the suitability of VIE for use as a marker in small, hemimetabolous insects. We found no evidence from our data that injecting termites with VIE affected survival. Our data also revealed no large changes in behavior in VIE‐injected termites—we did find evidence that treatment individuals allogroomed less and antennated toward others more than controls overall and that treatment individuals performed less trophallaxis than control individuals on day 10, however, these effects were very small. We were able to confirm that VIE marks persist after molting and suggest that, with appropriate caution, VIE could be used as a method to individually mark small insects like termites.

While we observed no statistically significant difference in survival between treatment and control groups, we acknowledge that there was an increase in the hazard among treatment termites, such that VIE‐injected individuals were approximately twice as likely to die at any given time compared to control individuals. This seemingly large hazard ratio could be driven by the small number of observed deaths in both groups (and the resulting wide 95% CI). Our post hoc simulations revealed that in 60% of trials, we would expect to observe a significant difference in survival between treatment and control groups, given the hazard ratio that we found. This suggests that there could be an effect of using VIE on survival that we did not detect in our experiment. All methods of marking insects are likely to cause some disturbance (Hagler & Jackson, 2001), and the increase in hazard that is deemed acceptable for a given study will depend on both ethical and practical considerations of specific research questions. It is important that harm is limited to maintain ethical standards and public support, as well as scientific validity (Drinkwater et al., 2019; Freelance, 2019).

We found evidence that individuals in the treatment group allogroomed less and antennated more than those in the control group (across all observation days). Allogrooming is associated with pathogen resistance in termites (Rosengaus et al., 1998). A reduction in this type of behavior could indicate an energetic cost to the VIE implant. Increased antennation in treatment individuals could indicate that they were more stimulated by their social environment than controls as antennation has been associated with identity‐checking and other social communication in termites (Thompson et al., 2020). However, we did not find a difference in the level of manipulation of the (abiotic) environment between treatment and control groups, suggesting that there was no general increase in response to external stimuli. The differences in both allogrooming and antennation were qualitatively very small (+0.02 and +0.0004, respectively), suggesting that the observed effect could have been due to differences between the two groups that were not accounted for by the random effects included in the model, or by a variable that we did not measure. There is potential for our result to be explained by observer bias since we were unable to collect behavioral data without revealing treatment to the observer. Behavioral differences between control and treatment groups could be negated by using VIE across all individuals (leaving no individuals unmarked) because location and color combinations provide opportunity to uniquely mark hundreds of individuals.

We found that treatment individuals performed less trophallaxis on day 10 than controls. Trophallaxis is thought to play a role in suppression of caste differentiation (Korb & Schmidinger, 2004), which is likely impacted by separation from the royal pair, as, under these circumstances, pseudergates (totipotent termite helpers) can differentiate into a reproductive form (Johns et al., 2009). If VIE implants incur an energetic cost, control individuals might be expected to engage in more competitive behavior than treatment individuals, such as suppressing the development of others into reproductive forms. However, this effect was small (−0.00012) and our experiment also revealed increased allogrooming and butting behavior in both treatment and control termites at day 10 post‐treatment. Increased allogrooming and butting behavior in lower termites is associated with reproductive disinhibition, particularly in orphaned colonies, when pseudergates can become reproductive (Hoffmann & Korb, 2011; Johns et al., 2009; Penick et al., 2013). That butting and allogrooming increased for both treatment and control individuals suggests that both groups responded to being separated from the colony.

The behavioral differences that we found in both treatment and control groups on day 10 of this study raise a broader issue about the effect of artificial experimental conditions on behavior. Behavioral changes in termites have been reported following disturbance and periods in laboratory culture, and behavior can differ in tests of similar phenomena (e.g., nestmate recognition) depending on assay design (Cornelius & Osbrink, 2009). Similarly, behavioral changes are reported following separation of pseudergates from reproductives (Konishi & Matsuura, 2021; Penick et al., 2013), but the effects of separation from reproductives on survival of pseudergates are unknown. Taken together, this suggests that termites are sensitive to disturbance and that caution should be taken when drawing general conclusions about behavior and causes of death in studies that disrupt termites, for example by moving them into an unfamiliar arena away from their natal colony.

We observed two instances in which VIE‐injected termites molted, and we were able to confirm in these cases that VIE marks persisted after this process, demonstrating that VIE represents an effective marking application for termites and potentially other hemimetabolous species. However, we observed six VIE‐injected individuals that lost their marks during this study. In these cases, individuals were not noticeably paler, nor did we find any shed exoskeletons in their housing, suggesting that mark loss may not be due to molting. Since we recorded instances of molting from videos it is possible that these individuals did molt, and that their mandibles and exoskeleton sclerotized, and their shed exoskeleton was consumed by colony mates, between video recordings; although by this line of reasoning, it is also likely that our observation of molting across treatment groups is an under‐estimate and more VIE‐injected individuals than the two we directly observed molted without losing their mark. A plausible explanation for mark loss is that there could have been inconsistencies in application. For example, if a smaller amount of elastomer was injected, or if the elastomer was not injected deep enough, it could be more readily ejected or broken down by the termite immune system, resulting in the loss of the mark. Altering specific methods of application—for example, using an automatic rather than manual syringe—could help to remove these possible inconsistencies and improve the reliability of the VIE marking.

## 5 CONCLUSIONS

Our findings suggest that, if used with appropriate caution, VIE could be a useful method to mark termites, and potentially other hemimetabolous insects, in experimental studies. We found that the changes in survival and behavior were small, suggesting that VIE might be appropriate in termites given that care is taken to minimize other sources of disturbance. Extensions of trials similar to this one would facilitate better understanding of the long‐term and physiological effects of VIE on marked termites. More broadly, VIE could be successfully used as a method of marking other hemimetabolous insects because the marks appear to be persistent through molts—a necessary feature of marking these species. Further studies in a range of other hemimetabolous insects would help establish VIE as a viable and widely applicable marking method.

## CONFLICT OF INTEREST

None declared.

## AUTHOR CONTRIBUTION

**Rebecca F. B. Padget:** Data curation (lead); Formal analysis (lead); Investigation (equal); Methodology (equal); Project administration (equal); Visualization (lead); Writing‐original draft (lead); Writing‐review & editing (supporting). **Faye J. Thompson:** Conceptualization (lead); Data curation (supporting); Formal analysis (supporting); Funding acquisition (lead); Investigation (equal); Methodology (equal); Project administration (equal); Supervision (lead); Visualization (supporting); Writing‐original draft (supporting); Writing‐review & editing (lead).

## Supporting information

Supplementary Material

## Data Availability

Data used in this paper are available on Dryad (https://doi.org/10.5061/dryad.fn2z34tv3). Code for statistical models is available at https://github.com/beckypadget/vie‐trials.
