# Supplementary material for: Marking through molts: An evaluation of visible implant elastomer to permanently mark individuals in a lower termite species
Source: Ecol Evol. 2021 Aug 18;11(18):12834–44. doi: 10.1002/ece3.8030 (PMC8462160; doi:10.1002/ece3.8030)
Supplement: Supplementary file 1 — Supplementary Material [file ECE3-11-12834-s001.docx]

Marking through molts: an evaluation of visible implant elastomer to permanently mark individuals in a lower termite species

**Supporting Information**

**Table S1: The number of individual termites (out of the group of five) that died in each treatment group from each colony.
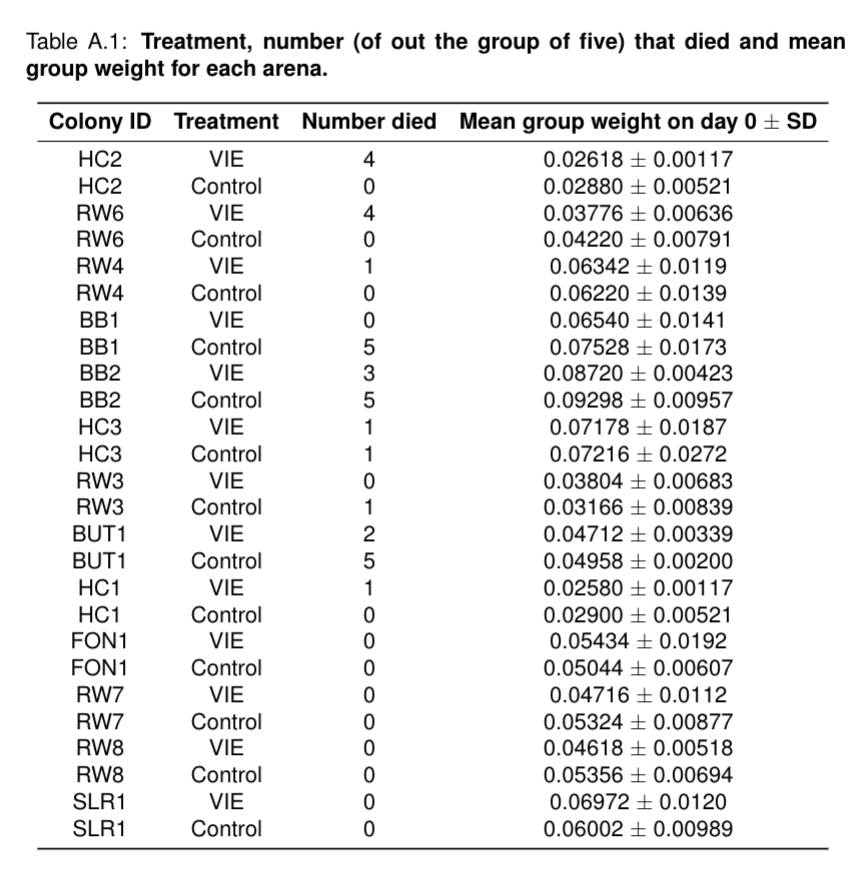
**


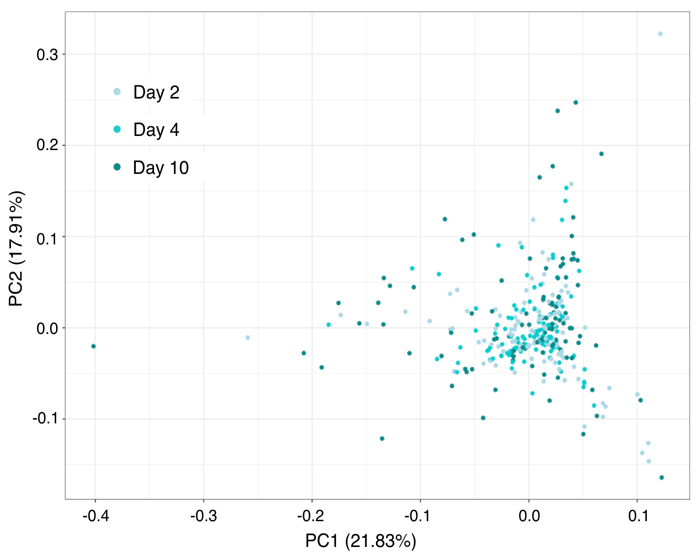

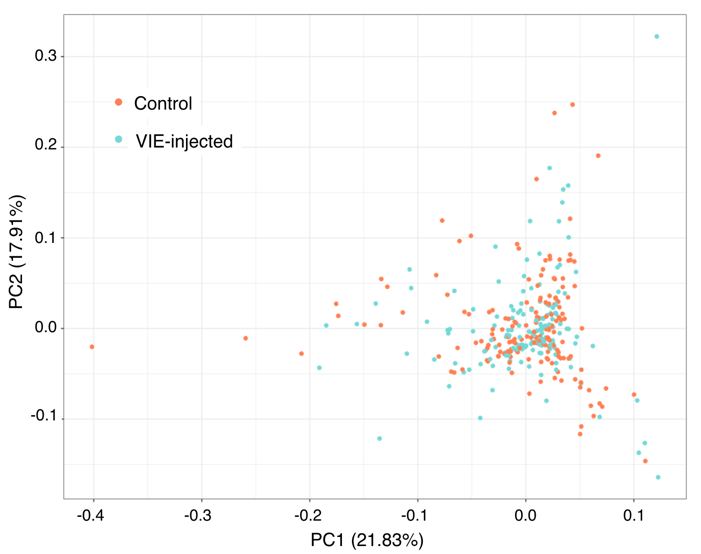


**Figure S1: Principal component analysis showed that behaviors did not cluster by either treatment (a) or day (b).** Principal components also did not account well for variation across behaviors, and the first two principal components explained only 21.82% and 17.91% of the variation suggesting no common underlying behavioral drivers.


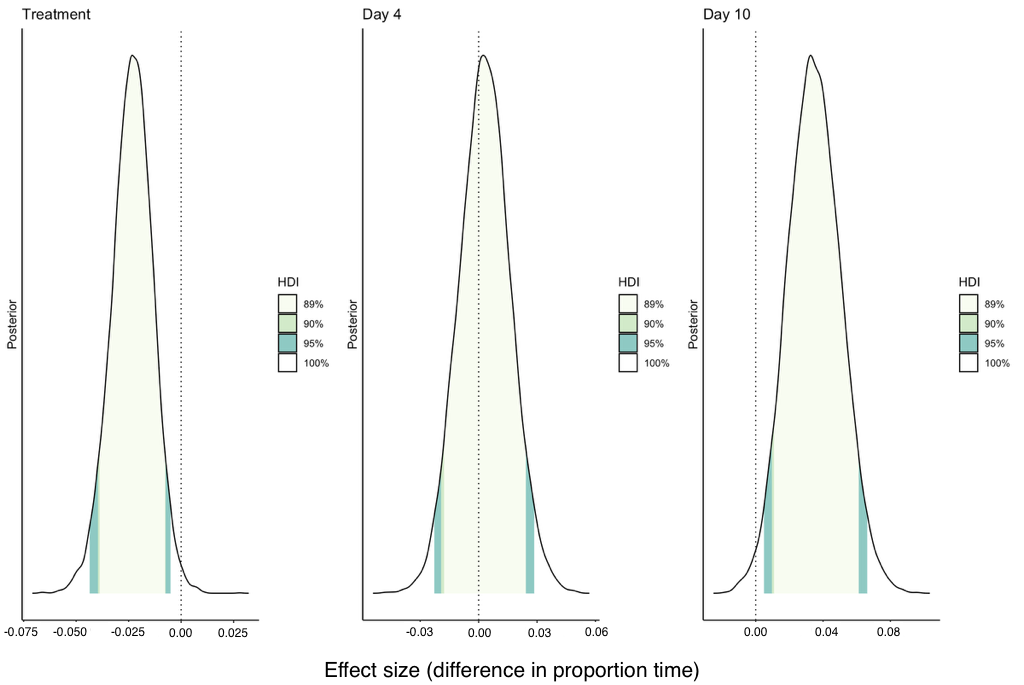


**Figure S2: Posterior distributions of predicted effect sizes for each predictor in the allogrooming model.** Regions spanning 89%, 90%, and 95% highest posterior density intervals (HDI) are shown.


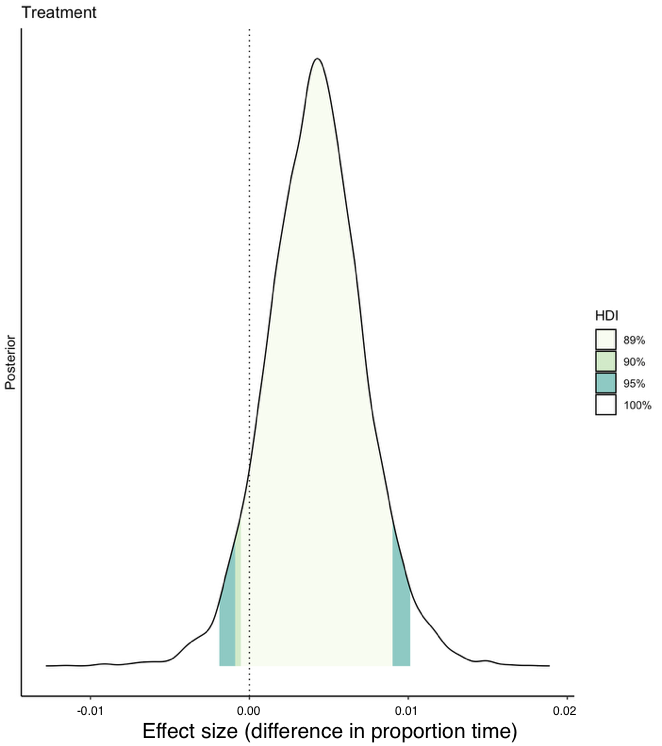


**Figure S3: Posterior distributions of predicted effect sizes for the treatment predictor in the model of antennation.** Regions spanning 89%, 90%, and 95% highest posterior density intervals (HDI) are shown.


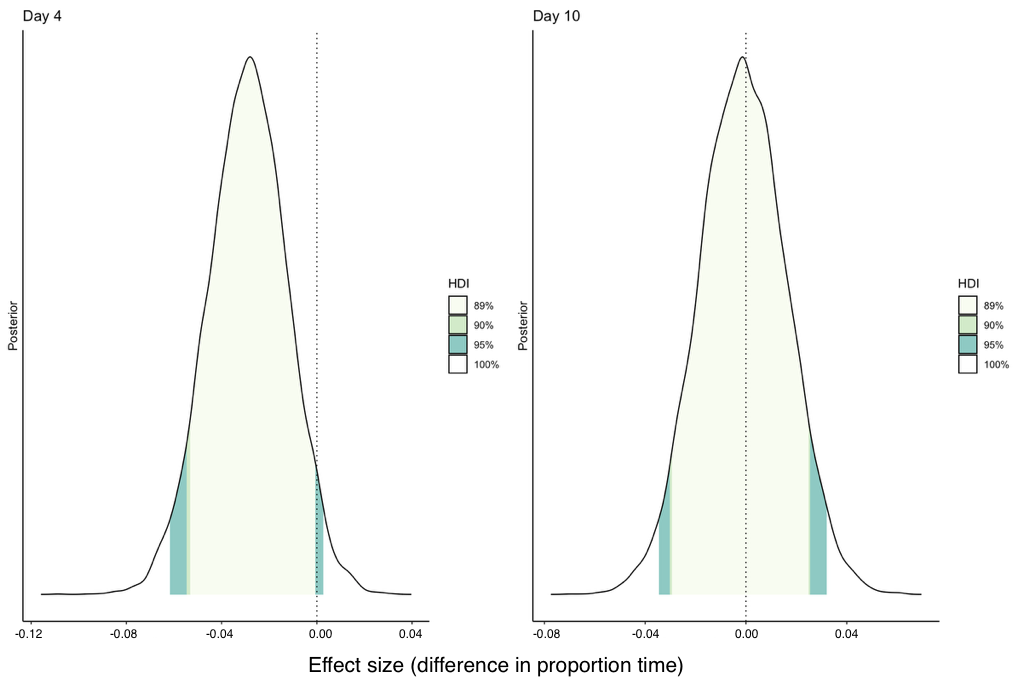


**Figure S4: Posterior distributions of predicted effect sizes for the day predictors in the model of environment manipulation.** Regions spanning 89%, 90%, and 95% highest posterior density intervals (HDI) are shown.


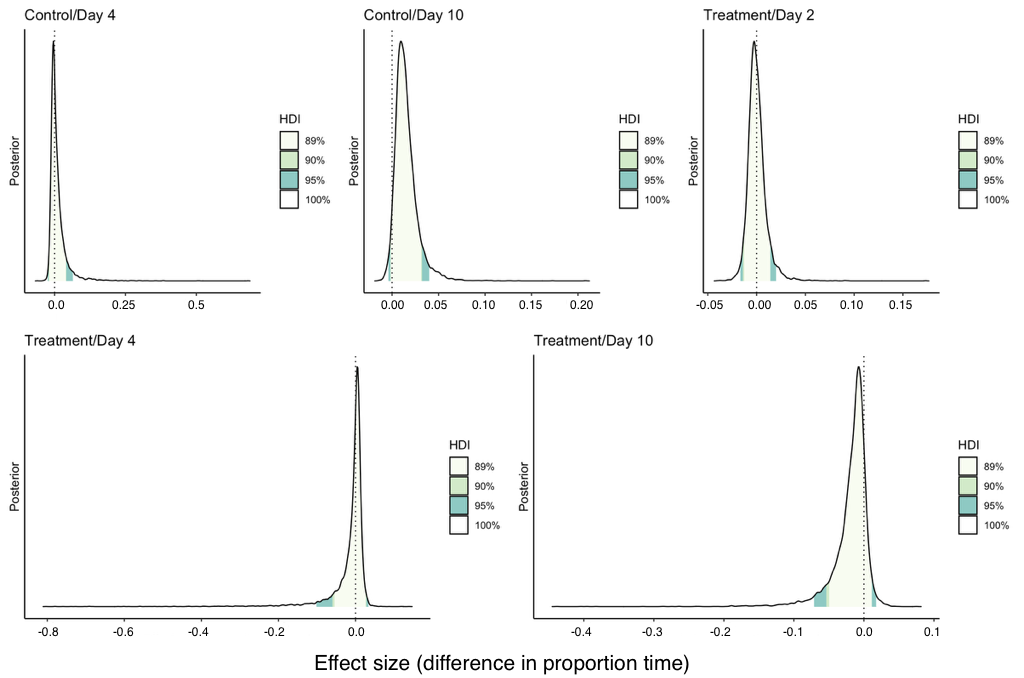


**Figure S5: Posterior distributions of predicted effect sizes for all predictors in the model of trophallaxis.** Regions spanning 89%, 90%, and 95% highest posterior density intervals (HDI) are shown.


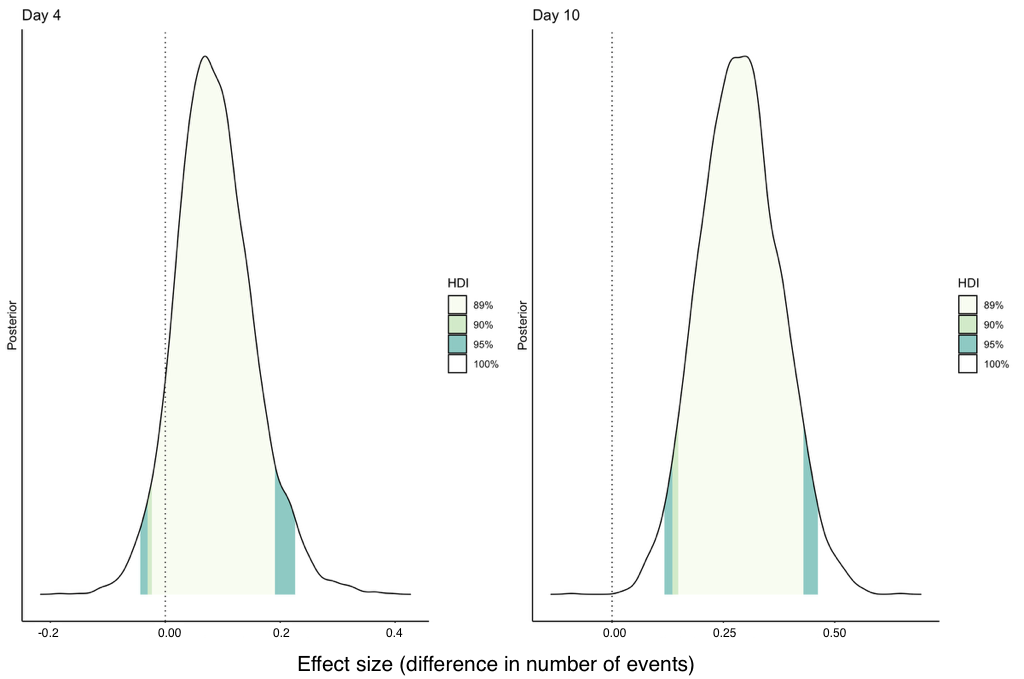


**Figure S6: Posterior distributions of predicted effect sizes for the day predictors in the model of butting.** Regions spanning 89%, 90%, and 95% highest posterior density intervals (HDI) are shown.


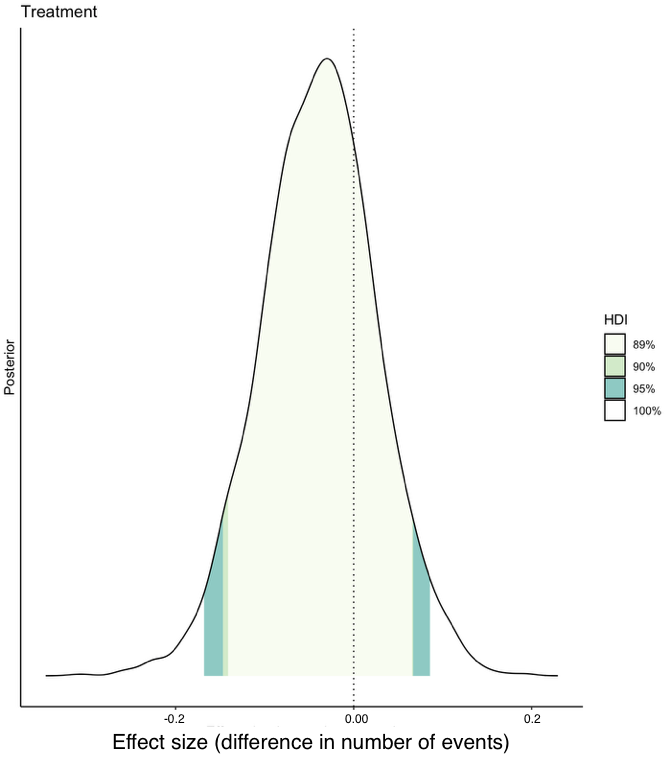


**Figure S7: Posterior distributions of predicted effect sizes for the treatment predictor in the model of self-grooming.** Regions spanning 89%, 90%, and 95% highest posterior density intervals (HDI) are shown.


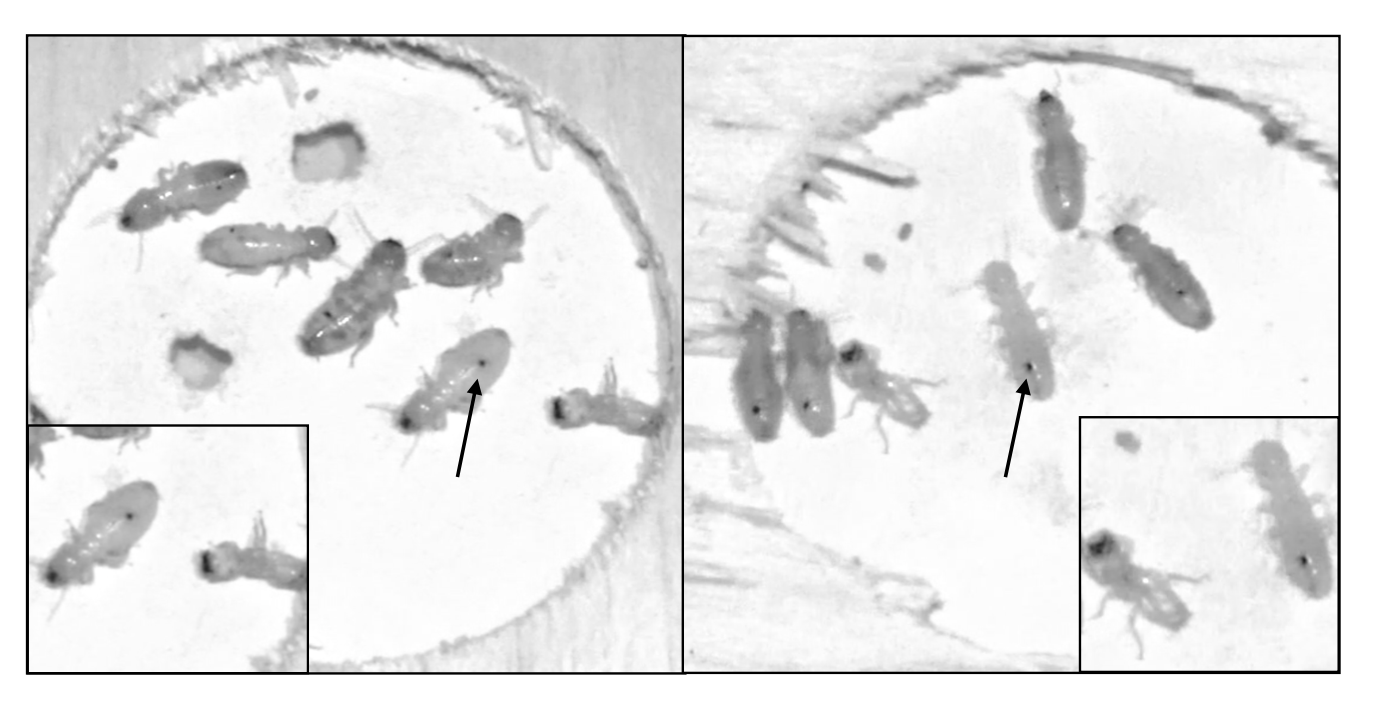


**(a)**

**(b)**

**Figure S8: Visible implant elastomer marks are retained after molting.** Images show the two cases of observed molting in two separate treatment groups (a) and (b). Each image shows a capture from a video of the entire treatment group. Circles indicate the molted termite, which is noticeably paler, and its shed exoskeleton. The arrows indicate the VIE mark that is still visible on the molted individuals. Square insets show a close-up image of the molted individual with its exoskeleton and visible VIE mark.
